# Prevalence and Virulence of Commensal Pseudomonas Aeruginosa Isolates from Healthy Individuals in Southern Vietnam (2018–2020)

**DOI:** 10.3390/biomedicines11010054

**Published:** 2022-12-26

**Authors:** Nguyen Bao Vy Tran, Quang Minh Truong, Lam Que Anh Nguyen, Ngoc My Huong Nguyen, Quang Hung Tran, Thi Tuyet Phuong Dinh, Vinh Son Hua, Van Dung Nguyen, Peter A. Lambert, Thi Thu Hoai Nguyen

**Affiliations:** 1 School of Biotechnology, International University, Vietnam National University, Ho Chi Minh City 700000, Vietnam; 2 College of Health and Life Sciences, Aston University, Birmingham B4 7ET, UK; 3 Research Center for Infectious Diseases, International University, Vietnam National University, Ho Chi Minh City 700000, Vietnam

**Keywords:** colonization risk factors, commensal *Pseudomonas aeruginosa*, sinusitis, virulence

## Abstract

Understanding the colonization of *Pseudomonas aeruginosa* (*P. aeruginosa*) in healthy humans is useful for future prevention and treatment of *P. aeruginosa* infection. This study aimed to investigate the prevalence and risk factors of of *P. aeruginosa* colonization in healthy humans. At the same time, the virulence of the isolated *P. aeruginosa* was also studied. In the study, 609 Vietnamese volunteers (310 females and 299 males, age range of 2 to 73 years), who had no acute infection or disease symptoms participated at the time of sample collection. Samples were taken from the throat, nostrils, and outer ears. *P. aeruginosa* was found in 19 participants (3.12%, 95% CI: 0.017–0.045), mainly from the throat (11/19, 57.89%). Participants with a history of sinusitis were 11.57 times more likely to be colonized with *P. aeruginosa* than participants without a history of sinusitis (OR: 11.57, 95% CI: 4.08–32.76, *p*-value < 0.0001, Fisher’s Exact test). Age and sex were not significantly associated with *P. aeruginosa* colonization. Among 16 *P. aeruginosa* isolates used in virulence tests, 100% (16/16) were positive for the synthesis of biofilm, pyocyanin, and siderophores; 93.75% (15/16) isolates were positive for the synthesis of gelatinase and protease; and 50% (8/16) isolates were positive for lipase. There were no differences in the pattern and range of virulence factors of *P. aeruginosa* isolates taken from participants with and without sinusitis history. *P. aeruginosa* colonized 3.12% of participants, and its presence was associated with sinusitis history.

## 1. Introduction

*P. aeruginosa* is a Gram-negative bacterium popular in soil, water, and moist areas. It is also a part of the normal flora of humans, which is isolated on the skin (0–2%), in the throat (0–3.3%), and in the stool (2.6–24%) [1]. Under continuous pressure from the human immune system, commensal *P. aeruginosa* can transform into a virulent pathogen [2]. *P. aeruginosa* is classified as an opportunistic pathogen that can cause serious infections, particularly in immunocompromised patients, such as in cystic fibrosis, and patients with severe burns [3]. With numerous extracellular enzymes, this bacterium easily adapts to persist, replicate, and attack the host [4]. Extracellular enzymes such as protease, gelatinase, and lipase damage the proteins of host cells, interfere with the immune response, delay the wound-healing process, and are associated with motility, biofilm architecture, and rhamnolipid production [5,6,7,8]. 

Besides extracellular enzymes, this bacterium has many other virulence factors, such as biofilm, pyocyanin, siderophores, protease, gelatinase, and lipase [9,10]. Biofilm is a complex structure that contains many planktonic cells sticking together by extracellular polymeric substances [11]. This structure can form on virtually any moist surface, such as living tissues, medical devices, water pipes, and so on. Furthermore, biofilm contributes to the persistence of *P. aeruginosa* infection by protecting this pathogen against the host defenses and antimicrobial strategies [12]. Cells within the biofilm grow slowly, which makes antibiotics work ineffectively [13]. Pyocyanin is a blue phenazine pigment secreted by *P. aeruginosa* [14]. It damages the host by involving in oxidation-reduction reactions. The oxidants from pyocyanin cause cell respiration dysfunction, calcium homeostasis disruption, and so on [15]. Moreover, pyocyanin possesses antimicrobial properties, so *P. aeruginosa* can use it as a weapon to kill other microorganisms and become predominant at the site of the infection [16]. Siderophores are low molecular-weight peptides that have the ability to chelate and deliver iron to bacterial cells. Although iron is crucial for all living organisms, including bacteria, the insoluble iron (Fe^3+^) is abundant in nature. This makes the uptake of iron by *P. aeruginosa* difficult [17]. Besides scavenging iron, they also stimulate biofilm formation [18]. In addition, siderophore-iron complexes can induce inflammation and oxidative damage, which increase the virulence of *P. aeruginosa* [19]. 

The colonization of commensal bacteria like *P. aeruginosa* on healthy humans deserves special attention because it can be a potential factor for subsequent disease [20]. Because it is opportunistic, it can cause an outbreak by being transmitted among healthy individuals before being detected [21]. So far, studies on *P. aeruginosa* colonization and its virulence, especially in the Vietnamese population, are limited. It is unclear whether sex, age, or any medical history affect the presence of *P. aeruginosa* in our body. Our study was the first study to analyze the prevalence of commensal *P. aeruginosa* in Southern Vietnam, its ability to produce common virulence factors, and the associated factors with *P. aeruginosa* colonization.

## 2. Materials and Methods

### 2.1. Commensal P. aeruginosa Isolation

From 2018 to 2020, throat, naris, and outer ear swab samples of Vietnamese people living in the Southeast area were collected and cultured on *Pseudomonas* selective Cetrimide media. This study was approved by the Ethics Committee of Vietnam National University of Ho Chi Minh City (Date 6 June 2019, No 1007/DHQG-KHCN). 

These areas were chosen for sample collection because of their humidity and sampling convenience with less discomfort for the participants. All volunteers or volunteers’ guardians gave their signed informed consent. Only people with no declared current acute infection or disease symptoms were included in the study. Colonies obtained on Cetrimide agar were further characterized using a Gram-staining, polymerase chain reaction (PCR) with *oprL* primers [22] as a screening step, and *16S rRNA* sequencing for confirmation. For further experiments, all the *P. aeruginosa* isolates were stored in Luria Bertani broth containing 30% glycerol at −80 °C.

### 2.2. P. aeruginosa Identification

#### *oprL*—Specific Polymerase Chain Reaction and *16S rRNA* Sequencing

The specific *oprL* primers were used to primary detect *P. aeruginosa* (forward: 5′-ATGGAAATGCTGAAATTCGGC-3′ and reverse: 5′-CTTCTTCAGCTCGACGCGACG-3′) (PHUSA GENOMICS, Can Tho City, Vietnam) and followed the previous study [22]. All positive isolates for *oprL* were sent to NAM KHOA Biotek Company (Ho Chi Minh City, Vietnam) for *16S rRNA* sequencing. The confirmed *P. aeruginosa* isolates were used for further experiments.

### 2.3. Virulence Testing

Biofilm, pyocyanin, siderophores, lipase, protease, and gelatinase of the isolated *P. aeruginosa* isolates were analyzed. *P. aeruginosa* ATCC 9027 (American Type Culture Collection, Manassas, VA, USA) was used as a positive control for all the producing virulence tests because it was a non-virulent strain isolated from the human outer ear by the American Type Culture Collection and it has ability to produce tested virulence factors in this study [23,24,25,26].

#### 2.3.1. Biofilm

Biofilm production of commensal *P. aeruginosa* isolates was tested by the crystal violet (HiMedia Laboratories, Kennett Square, PA, USA) staining method. The optimal density (OD) was measured at 550 nm by BioTek Synergy HTX Multimode Reader (Agilent, Santa Clara, CA, USA) [18]. Commensal isolates were classified into negative, weak, moderate and strong biofilm producers by following this category: OD ≤ ODc (negative); ODc < OD ≤ 2 × ODc (weak); 2 × ODc < OD ≤ 4 × ODc (moderate); and OD > 4 × ODc (strong), in which ODc was the cut-off value (ODc = OD negative control + 3 × its standard deviation). OD was the optimal density of *P. aeruginosa* isolates [27].

#### 2.3.2. Pyocyanin

Pyocyanin was extracted and measured using the chloroform method [20]. *P. aeruginosa* isolate was cultured in glycerol-alanine (Gly-Ala) (HiMedia Laboratories, Kennett Square, PA, USA; Xilong Scientific Co., Ltd., Shantou, China) broth to maximize the yield of pyocyanin production in commensal isolates. When the OD_600 nm_ bacteria reached 0.08–0.1, 1% of the culture was inoculated in 5 mL of Gly-Ala broth at 37 °C for 24 h with shaking condition. After that, the supernatant was collected (centrifuge 6000 rpm for 15 m). A quantity of 3 mL of supernatant was mixed vigorously with 1.8 mL of chloroform (VN-CHEMSOL Co., Ltd., Ho Chi Minh City, Vietnam). Then, blue layer was collected and mixed with 0.2 N HCl (2:1). The top red color was collected and measured at 520 nm using BioTek Synergy HTX Multimode Reader (Agilent, Santa Clara, United States). The concentration of pyocyanin (μg/mL) was estimated by multiplying the OD at 520 nm by 17.072 (the molar extinction coefficient of pyocyanin 520 nm) [28,29]. 

#### 2.3.3. Siderophores, Lipase, Protease, and Gelatinase

Before agar plate test, all tested isolates and controls were cultured in Luria-Bertani broth for 24 h at 37 °C. After the OD_600 nm_ reached the range 0.08–0.1, 5 µL of the cultures spotted on CAS blue agar [30], tributyrin agar (1% tributyrin), skim milk (3% skim milk, Brain Heart Infusion (BHI) medium), or gelatin agar (8% gelatin, BHI) for detecting siderophores, lipase, protease, and gelatinase, respectively. The enzymatic activity (EA) was estimated by measuring the halo zone size: EA = (D − d)/2, where D was the diameter of clear zone (mm) and d was the diameter of a colony (mm). It was categorized as negative (0 mm), weak (<2 mm), moderate (2–4 mm), and strong (>4 mm) activity [31]. Besides *P. aeruginosa* ATCC 9027, *Staphylococcus aureus* (*S. aureus*) was used a positive control for protease and lipase tests, while *Vibrio cholerae* (*V. cholerae*) was used for gelatinase. In case of siderophores, *E. coli* was also used as a positive control, while *S. aureus* was a negative control because it was Gram-positive and could not survive on CAS blue agar [30]. All commercial materials for these tests were purchased from Sigma-Aldrich, St. Louis, Missouri, United States; HiMedia Laboratories, Kennett Square, United States; Xilong Scientific Co., Ltd., Shantou, China.

### 2.4. Data Analysis

Each experiment was performed in triplicate. IBM^®^ SPSS^®^ Statistics 20.0 (IBM, Armonk, New York, NY, USA) was used to analyze the data. Chi-square, Fisher’s Exact and Ordinal Regression tests were used to determine the risk factors for *P. aeruginosa* colonization. In addition, an ANOVA analysis was used to show the association between sinusitis history and commensal *P. aeruginosa* virulence. The *p*-value was set to be <0.05.

## 3. Results and Discussion

### 3.1. Prevalence of Commensal P. aeruginosa Isolates in Vietnamese Population

There were 612 volunteers who signed informed consent and provided samples. Three were excluded due to their declared health condition and 609 participants were finally included in the study. The number of male and female participants was similar, 299 males (49.10%) versus 310 (50.90%) females. Because the age of participants was not variety, it could only be grouped into three categories: young persons (0–17), adult (18–59), and older persons (≥60). It is well-noted that having sinusitis is a common health problem in the population. More than 1/5 of healthy declaring people experienced sinusitis. The summary of all participants and *P. aeruginosa* carrier characteristics was described in Table 1.

From 609 participants, 35 *P. aeruginosa*-like isolates were obtained. These isolates grew on cetrimide selective media, being Gram-negative rod shaped and positive for *oprL*. The *oprL* gene encodes the outer membrane peptidoglycan-associated lipoprotein, which has an important role in the interaction between bacteria and environment, is a common and useful target for *Pseudomonas* detection [22,32,33].

A total of 20 out of 35 isolates positive for *oprL* (57.14%) were confirmed as *P. aeruginosa* via *16S rRNA* sequencing (Supplementary Table S1). Among 20 *P. aeruginosa* isolates, two isolates came from one volunteer (from throat and nostrils) and 18 others came from a single site on each participant. In summary, 19 out of 609 participants (3.12%, 95% CI: 0.017–0.045) were colonized with *P. aeruginosa* (Supplementary Table S2). This was in agreement with previous data showing that *P. aeruginosa* was not commonly detected in healthy people, but under conditions of antibiotic exposure or hospitalization its prevalence, mainly in the throat and stool, was increased [34,35]. For example, in the case of bronchiectasis, which is a chronic respiratory disease associated with *P. aeruginosa*, the prevalence of *P. aeruginosa* colonization in bronchiectasis patients ranged from 9% to 33% [36]. Additionally, Casetta et al. also found that 17.5% of pregnant women were colonized by *P. aeruginosa* after more than 48 h hospital admission [37]. Importantly, colonization with *P. aeruginosa* represents a risk of infection: Gómez-Zorrilla et al. reported that 43% of colonized patients in their study developed infection [38]. 

From our findings, *P. aeruginosa* isolates were likely obtained from the throat (11/20 *P*. *aeruginosa* isolates), while *P. stutzeri* isolates were rather from the nostril area (9/12 *P. stutzeri* isolates). Furthermore, the throat was also primarily colonized by *P. aeruginosa* (11/13 throat-derived isolates from the throat, 84.62%). Only one *P. azelaica*, one *P. nitroreducens* isolate and no *P. stutzeri* isolate were found in this area (Supplementary Table S3). It was consistent with previous studies in which *P. aeruginosa* is likely found in a mucoid and humid area and is the most common *Pseudomonas* causing infection in human [39,40].

### 3.2. Relationship between Sex and Age to P. aeruginosa Colonization

Among 19 commensal *P. aeruginosa* carriers, 63.16% (12/19) were female and 36.84% (7/19) were male. However, there were no associations between sex and *P. aeruginosa* colonization of healthy humans (OR: 1.68, 95% CI: 0.65–4.33, *p*-value = 0.29 (Fisher’s Exact Test) and Chi-Square: *p*-value = 0.278). To our knowledge, there is still a lack of information about the effect of sex on *P. aeruginosa* colonization in healthy humans. In contrast to our finding, the study of *P. aeruginosa* colonization from wounds and burns swabs of patients showed the higher rate of *P. aeruginosa* among the male than female samples, 55.6% and 44.4%, respectively [41]. However, the presence of *P. aeruginosa* was associated with a higher morbidity rate in females with respiratory dysfunction, such as cystic fibrosis and bronchiectasis. The estrogen in females could regulate the conversion of *P. aeruginosa* from non-mucoid to mucoid forms, which are believed to have higher virulence [42]. The relationship between sex and *P. aeruginosa* colonization is described in Table 2. 

Although the group 18–59 years had the highest number of carriers (84.21%, 16/19 *P. aeruginosa* carriers), age was not a significant factor for *P. aeruginosa* colonization (0–17: 65 participants, 18–59: 514 participants, and ≥60: 30 participants) (*p*-value = 0.448, Ordinal Regression). It was because the 95% CI include number 1 in both adult (3.11%, OR: 2.02, 95% CI: 0.26–15.43, *p*-value < 0.05, Fisher’s Exact test) and older person (6.67%, OR: 6.75, 95% CI: 0.59–77.55, *p*-value > 0.05, Fisher’s Exact test) groups. Currently, the association between age and *P. aeruginosa* colonization in healthy humans is unclear, but age > 55 years is considered to be an independent predictor for *P. aeruginosa* colonization in bronchiectasis patients [43]. The incidence of infections caused by opportunistic pathogens seems to increase significantly in older ages [44] and colonization could be a risk factor for infection [45]. 

### 3.3. Relationship of Sinusitis History to P. aeruginosa Colonization

Our data indicated that sinusitis is quite common in the population with 129/609 (21.18%) participants having sinusitis history (Table 1). Interestingly, participants with sinusitis history are more likely to be colonized with *P. aeruginosa* (14/129, 10.85%) compared to the ones without sinusitis history (5/480, 1.04%). It is estimated that the participants with sinusitis history have a risk of *P. aeruginosa* colonization 11.57 times higher than that of participants without sinusitis history (OR: 11.57, 95% CI: 4.08–32.76, *p*-value < 0.0001, Fisher’s Exact test, Table 2). Our data were somewhat in agreement with previous studies. For example, Niederman et al. indicated that the upper and lower respiratory tract was not colonized by Gram-negative bacteria under normal health conditions, but these sites could be harbored by these pathogens when illness developed [46]. In the case of cystic fibrosis patients, Shapiro et al. found that *P. aeruginosa* was detected in 38% of sinusitis-cystic fibrosis patients [47] while Kasper Aanæs et al., reported that the presence of *P. aeruginosa* in sinuses was associated with lung infection in cystic fibrosis patients [48]. However, data were also quite controversial as in some studies, *P. aeruginosa* was not a common pathogen in bacterial flora of chronic sinusitis patients, only present for 1% to 5% of cases [47,48,49]. 

### 3.4. Commensal P. aeruginosa Virulence

In this study, 16 out of 20 *P. aeruginosa* isolates were used for testing the production of key virulence factors, including biofilm, pyocyanin, siderophores, lipase, protease, and gelatinase (Supplementary Tables S4–S6). A total of 100% (16/16 *P. aeruginosa* isolates) of isolates were positive for biofilm, pyocyanin, siderophores and hemolysin; 93.75% (15/16) of isolates were positive for gelatinase and protease; and 50% (8/16) of isolates were positive for lipase (Table 3). 

All tested isolates showed the ability to produce biofilm (16/16). Among them, there were 25% (4/16) of weak, 56.25% (9/16) of moderate, and 18.75% (3/16) of strong biofilm producers. In addition, the biofilm production of commensal *P. aeruginosa* isolates was weaker than the *P. aeruginosa* ATCC 9027 (positive control). Some studies showed less dominance of strong biofilm producers in the clinical isolates [50] and isolates from cystic fibrosis patients [51]. Biofilm production did not associate with poor clinical outcomes [52]. Moreover, it was reported that *P. aeruginosa* often grew planktonically and did not form biofilm under laboratory conditions [53]. 

**Figure 1:**
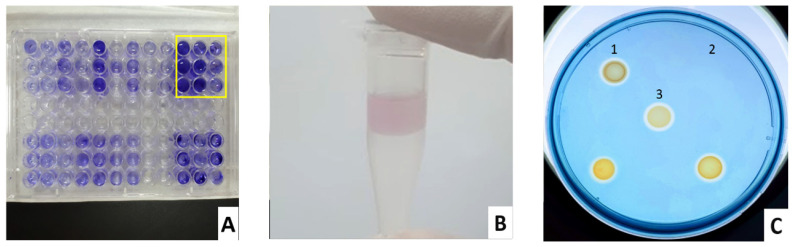
The production of biofilm (**A**), pyocyanin (**B**) red layer in 0.2 N HCl, and (**C**) siderophores of commensal *P. aeruginosa* isolates. (**A**): yellow rectangle—*P. aeruginosa* ATCC 9027; others—*P. aeruginosa* isolates; C1: *E. coli*, C2: *S. aureus*, C3: *P. aeruginosa* ATCC 9027; others—*P. aeruginosa* isolates. HDTMA in CAS agar was toxic to Gram-positive bacteria, so *S. aureus* was inhibited and did not grow at the C2 position.

All tested commensal *P. aeruginosa* isolates had the ability to secrete phenazine pyocyanin and siderophores (Figure 1, Table 3). High pyocyanin producers (>18 µg/mL) were positively correlated with septic shock [54]. In our study, the average pyocyanin concentration from commensal *P. aeruginosa* isolates was 0.61 µg/mL. For siderophore production, the ability of the isolates varied following the isolation sites depending on the availability of free iron. Clinical *P. aeruginosa* isolates from urinary tract infection produced more siderophores compared with burn skin [55]. In our study, the commensal isolates were unsurprisingly weak siderophore producers (Table 3).

**Figure 2:**
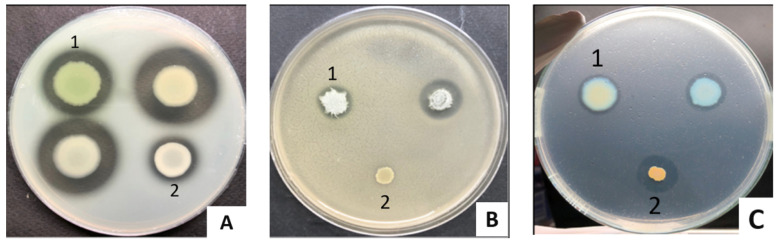
The production of protease (**A**), gelatinase (**B**), and lipase (**C**) of commensal *P. aeruginosa* isolates. A1, B1, C1: *P. aeruginosa* ATCC 9027; A2, B2: *S. aureus*; C2: *V. cholerae*; the others: *P. aeruginosa* isolates.

While 93.75% of the tested commensal *P. aeruginosa* produced protease and gelatinase, their average enzymatic activity was 0.28 ± 0.09 (mm) and 0.29 ± 0.06 (mm), respectively. Only 50% of tested isolates were positive for lipase, for which the average halo size was 0.18 ± 0.04 (mm) (Figure 2, Table 3). Our data are comparable with previous studies on clinical isolates from chronic leg ulcers where 91.67% produced protease, 66.67% produced gelatinase, and 83.33% produced lipase [56], indicating the common presence of these extracellular enzymes in all *P. aeruginosa* isolates.

There were no significant differences in virulence between *P. aeruginosa* isolates from carriers with and without sinusitis history (*p*-value > 0.05, ANNOVA test) (Supplementary Table S7). Sharna’s study showed that *P. aeruginosa* isolates from cystic fibrosis lung increased biofilm, alginate, and some virulence gene expression [57]. Furthermore, elastase was associated with chronic rhinosinusitis severity [58]. So far, there was no evidence on the increased virulence of commensal *P. aeruginosa* isolated from people with sinusitis history. However, people with a sinusitis history were more likely to be *P. aeruginosa* carriers than others, which is a potential risk of subsequent infection with *P. aeruginosa* [59], and *P. aeruginosa* infections were known to be particularly dangerous for patients with respiratory diseases [60,61]. 

## 4. Conclusions

For the first time, we revealed that the prevalence of commensal *P. aeruginosa* in the Southern Vietnamese population was 3.12%. Sinusitis could be a potential factor contributing to the colonization of *P. aeruginosa*. The commensal *P. aeruginosa* isolates can produce multiple virulence factors, including biofilm, pyocyanin, siderophores, lipase, protease, gelatinase and lipase. Further studies on the transition of *P. aeruginosa* from commensal to pathogenic state and its persistence in the carriers could give us deeper understanding on the host-pathogen interaction, which would be useful for prevention and treatment of *P. aeruginosa* infections. 

## Figures and Tables

**Table 1:** Characteristics of 609 participants in the study.

Characteristics		Number Overall (*n* = 609)
Sex	Male	299 (49.10%)
	Female	310 (50.90%)
Age	0–17	65 (10.67%)
	18-59	514 (84.40%)
	≥60	30 (4.93%)
Health status	With sinusitis history	129 (21.18%)
	Without sinusitis history	480 (78.82%)

**Table 2:** Odd ratio (OR) for sex, age, and health status differences in *P. aeruginosa* carriers.

Characteristics		*P. aeruginosa* Carriers	Non-*P. aeruginosa* Carriers	OR	*p*-Value (Fisher’s Exact Test)
Sex	Female (*n* = 310)	12 (3.87%)	298 (96.13%)	1.68	0.29
	Male (*n* = 299)	7 (2.34%)	292 (97.66%)	1.00	
Health status	With Sinusitis history (*n* = 129)	14 (10.85%)	115 (89.15%)	11.57	<0.0001
	Without sinusitis history (*n* = 480)	5 (1.04%)	475 (98.96%)	1.00	
Age groups, years	0–17 (*n* = 65)	1 (1.56%)	64 (98.46%)	1.00	
	18–59 (*n* = 514)	16 (3.11%)	498 (96.89%)	2.02	0.0496
	≥60 (*n* = 30)	2 (6.67%)	28 (93.33%)	6.75	0.125

Male, age group 0–17, and without sinusitis history were the reference category, hence their OR = 1.

**Table 3:** Production of biofilm, pyocyanin, siderophores, lipase, protease, and gelatinase in commensal *P. aeruginosa* isolates and *P. aeruginosa* ATCC 9027.

Virulence Factors	Average Virulence Value ± Standard Deviation	
	Commensal *P. aeruginosa* Isolates	*P. aeruginosa* ATCC 9027
Biofilm (OD_550 nm_)	0.11 ± 0.06	0.72 ± 0.10
Pyocyanin (µg/mL)	0.61 ± 0.40	0.60 ± 0.23
Siderophores (mm)	1.31 ± 0.34	1.33 ± 0.29
Lipase (mm)	0.18 ± 0.04	0.17 ± 0.03
Protease (mm)	0.28 ± 0.09	0.17 ± 0.03
Gelatinase (mm)	0.29 ± 0.06	0.22 ± 0.03

## Data Availability

All data generated or analyzed during this study are included in this published article and its supplementary information files.

## Supplementary Materials

The following supporting information can be downloaded at: https://www.mdpi.com/article/10.3390/biomedicines11010054/s1, Table S1: *Pseudomonas* species were isolated from three body sites (1-throat, 2-naris, 3-outer ear). They were conforming by 16S rRNA sequencing. The bold fonts indicate *P. aeruginosa* isolates; Table S2: List of 19 *P. aeruginosa* carriers; Table S3: *oprL*—positive *Pseudomonas* isolates and their colonization sites: Table S4: 16 commensal *P. aeruginosa* isolates were used in virulence tests; Table S5: Biofilm formation, pyocyanin, and siderophores in commensal *P. aeruginosa* isolates after inoculation at 37 °C for 24 h. The values were expressed as mean ± standard deviation; Table S6: Lipase, protease, and gelatinase values in commensal *P. aeruginosa* isolates after incubation at 37 °C for 24 h; Table S7: The production of tested virulence factors in commensal *P. aeruginosa* isolates from participants with and without sinusitis history. Average ± standard deviation.

## Abbreviations

*P. aeruginosa*: *Pseudomonas aeruginosa*; *P. stutzeri*: *Pseudomonas stutzeri*; *P. azelaica*: *Pseudomonas azelaica*; *P. nitroreducens*: *Pseudomonas nitroreducens*; *E. coli*: *Escherichia coli*; *S. aureus*: *Staphylococcus aureus*; ATCC, American Type Culture Collection; PCR: polymerase chain reaction; BHI: brain heart infusion; OR: odd ratio; OD: optical density; CAS: chrome azurol S; HDTMA: hexadecyltrimethylammonium bromide; SPSS: Statistical Package for the Social Sciences; ANOVA: Analysis of Variance.
