# Supplementary material for: Prevalence and Virulence of Commensal Pseudomonas Aeruginosa Isolates from Healthy Individuals in Southern Vietnam (2018–2020)
Source: Biomedicines. 2022 Dec 26;11(1):54. doi: 10.3390/biomedicines11010054 (PMC9855430; doi:10.3390/biomedicines11010054)
Supplement: Supplementary file 1 [file biomedicines-11-00054-s001.zip › biomedicines-2110176-supplementary.pdf]

Supplementary materials

Table S1. *Pseudomonas* species were isolated from three body sites (1-throat, 2-naris, 3-outer ear). Theywere conforming by *16S rRNA* sequencing. The bold fonts indicate *P. aeruginosa* isolates.

No.	Positive Isolates Positive for *oprL*	*16S rRNA* Sequencing
1	48.2	*P. stutzeri*
2	**113.1**	***P. aeruginosa***
3	138.2	*P. stutzeri*
4	154.3	*P. stutzeri*
5	156.2	*P. stutzeri*
6	175.2	*P. stutzeri*
7	176.2	*P. stutzeri*
8	182.2	*P. stutzeri*
9	**185.1**	***P. aeruginosa***
10	185.2	*P. stutzeri*
11	**211.3**	***P. aeruginosa***
12	228.2	*P. stutzeri*
13	229.3	*P. stutzeri*
14	**234.1**	***P. aeruginosa***
15	256.3	*P. azelaica*
16	**263.1**	***P. aeruginosa***
17	**267.1**	***P. aeruginosa***
18	303.1	*P. nitroreducens*
19	**306.3**	***P. aeruginosa***
20	**307.1**	***P. aeruginosa***
21	**307.2**	***P. aeruginosa***
22	**338.2**	***P. aeruginosa***
23	**355.1**	***P. aeruginosa***
24	386.2	*P. stutzeri*
25	**388.1**	***P. aeruginosa***
26	**464.3**	***P. aeruginosa***
27	**486.1**	***P. aeruginosa***
28	**499.2**	***P. aeruginosa***
29	**507.1**	***P. aeruginosa***
30	**544.2**	***P. aeruginosa***
31	565.3	*P. stutzeri*
32	**575.3**	***P. aeruginosa***
33	582.1	*P. azelaica*
34	**588.1**	***P. aeruginosa***
35	**600.2**	***P. aeruginosa***

Table S2. List of 19 *P. aeruginosa* carriers.

No.	Age Range (years)	Sex	Health Status
113	18-59	F	Sinusitis history
185	18-59	F	No sinusitis history
211	≥ 60	F	Sinusitis history
234	18-59	F	Sinusitis history
263	18-59	F	Sinusitis history
267	18-59	F	No sinusitis history
306	18-59	F	Sinusitis history
307	18-59	F	Sinusitis history
338	18-59	M	Sinusitis history
355	≥ 60	F	Sinusitis history
388	18-59	M	No sinusitis history
464	18-59	M	Sinusitis history
486	18-59	F	Sinusitis history
499	18-59	F	No sinusitis history
507	18-59	F	Sinusitis history
544	18-59	M	Sinusitis history
575	<18	M	Sinusitis history
588	18-59	M	No sinusitis history
600	18-59	M	Sinusitis history

Table S3. *oprL*- positive *Pseudomonas* isolates and their colonization sites.

Species	Total number (n = 35)	Throat (n = 13)	Naris (n = 14)	Outer ears (n = 8)
P. aeruginosa	20 (57.14%)	11 (84.62%)	5 (35.71%)	4 (50%)
P. stutzeri	12 (34.29%)	0	9 (64.29%)	3 (37.50%)
P. azelaica	2 (5.71%)	1 (7.62%)	0	1 (12.50%)
P. nitroreducens	1 (2.86%)	1 (100%)	0	0

Table S4. 16 commensal *P. aeruginosa* isolates were used in virulence tests.

No.	Commensal *P. aeruginosa* Isolates
1	113.1
2	185.1
3	211.3
4	234.1
5	263.1
6	267.1
7	306.3
8	307.1
9	307.2
10	338.2
11	355.1
12	388.1
13	464.3
14	486.1

15	499.2
16	507.1

Table S5. Biofilm formation, pyocyanin, and siderophores in commensal *P. aeruginosa* isolates after inoculation at 37°C for 24 hours. The values were expressed as mean ± standard deviation.

Samples	Biofilm (OD 550nm)	Pyocyanin (µg/mL)	Siderophores (mm)
113.1	0.05 ± 0.00	0.10 ± 0.04	1.00 ± 0.00
185.1	0.01 ± 0.01	0.73 ± 0.21	1.08 ± 0.14
211.3	0.13 ± 0.05	0.14 ± 0.10	1.08 ± 0.14
234.1	0.02 ± 0.00	0.05 ± 0.03	0.75 ± 0.25
263.1	0.09 ± 0.03	0.17 ± 0.03	1.08 ± 0.38
267.1	0.02 ± 0.01	1.22 ± 0.12	1.25 ± 0.25
306.3	0.16 ± 0.04	0.47 ± 0.23	1.25 ± 0.00
307.1	0.11 ± 0.05	0.50 ± 0.11	1.33 ± 0.14
307.2	0.10 ± 0.02	0.84 ± 0.15	1.41 ± 0.38
338.2	0.12 ± 0.07	0.71 ± 0.13	1.58 ± 0.14
355.1	0.15 ± 0.03	0.69 ± 0.13	1.83 ± 0.14
388.1	0.12 ± 0.06	0.22 ± 0.14	1.25 ± 0.25
464.3	0.21 ± 0.02	1.19 ± 0.17	2.17 ± 0.29
486.1	0.21 ± 0.06	1.28 ± 0.34	1.17 ± 0.14
499.2	0.14 ± 0.05	0.78 ± 0.33	1.17 ± 0.14
507.1	0.17 ± 0.01	0.70 ± 0.15	1.50 ± 0.25
P. aeruginosa ATCC 9027 (positive control)	0.72 ± 0.10	0.60 ± 0.23	1.33 ± 0.29

Table S6. Lipase, protease, and gelatinase values in commensal *P. aeruginosa* isolates after incubation at 37°C for 24 hours.

Samples	Lipase (mm)	Protease (mm)	Gelatinase (mm)
113.1	0.12	0.30	0.32
185.1	0.17	0.18	0.22
211.3	0.10	0.33	0.38
234.1	0.13	0.00	0.00
263.1	0.13	0.22	0.23
267.1	0.12	0.18	0.25
306.3	0.17	0.17	0.22
307.1	0.17	0.20	0.32
307.2	0.17	0.33	0.32
338.2	0.12	0.28	0.32
355.1	0.13	0.35	0.35
388.1	0.17	0.37	0.28
464.3	0.23	0.43	0.30
486.1	0.08	0.27	0.27
499.2	0.20	0.22	0.22
507.1	0.17	0.38	0.30
P. aeruginosa ATCC 9027 (positive control)	0.17	0.17	0.22

Table S7. The production of tested virulence factors in commensal *P. aeruginosa* isolates from participants with and without sinusitis history. Average ± standard deviation.

Virulence Factors	With Sinusitis History	Without Sinusitis History	*p*-value (ANOVA)
Biofilm (OD_{550nm})	0.13 ± 0.06	0.20 ± 0.3	0.39
Pyocyanin (ug/ml)	0.57 ± 0.41	0.71 ± 0.36	0.52
Siderophores (mm)	1.35 ± 0.39	1.22 ± 0.09	0.48
Lipase (mm)	0.14 ± 0.04	0.17 ± 0.03	0.27
Gelatinase (mm)	0.28 ± 0.1	0.24 ± 0.03	0.40
Protease (mm)	0.27 ± 0.11	0.22 ± 0.08	0.41
